# Fabrication of High Performance PVDF Hollow Fiber Membrane Using Less Toxic Solvent at Different Additive Loading and Air Gap

**DOI:** 10.3390/membranes11110843

**Published:** 2021-10-29

**Authors:** Hazirah Syahirah Zakria, Mohd Hafiz Dzarfan Othman, Siti Hamimah Sheikh Abdul Kadir, Roziana Kamaludin, Asim Jilani, Muhammad Firdaus Omar, Suriani Abu Bakar, Juhana Jaafar, Mukhlis A. Rahman, Huda Abdullah, Mohd Hafiz Puteh, Oulavanh Sinsamphanh, Muhammad Ayub

**Affiliations:** 1 Advanced Membrane Technology Research Centre (AMTEC), Universiti Teknologi Malaysia, Skudai 81310, Johor, Malaysia; hazirahzakria@gmail.com (H.S.Z.); roziana.kamaludin7@gmail.com (R.K.); juhana@petroleum.utm.my (J.J.); mukhlis@petroleum.utm.my (M.A.R.); ayub1977@graduate.utm.my (M.A.); 2 School of Chemical and Energy Engineering, Faculty of Engineering, Universiti Teknologi Malaysia (UTM), Skudai 81310, Johor, Malaysia; 3 Institute of Pathology, Laboratory and Forensics (I-PPerForM), Faculty of Medicine, Universiti Teknologi MARA (UiTM), Cawangan Selangor, Sungai Buloh 47000, Selangor, Malaysia; 4 Center of Nanotechnology, King Abdul-Aziz University, Jeddah 21589, Saudi Arabia; asim.jilane@gmail.com; 5 Scientific Computing and Instrumentation (SCnI) Research Group, Physics Department, Faculty of Science, Universiti Teknologi Malaysia, Skudai 81310, Johor, Malaysia; firdausomar@utm.my; 6 Nanotechnology Research Centre, Faculty of Science and Mathematics, Universiti Pendidikan Sultan Idris, Tanjung Malim 35900, Perak, Malaysia; suriani@fsmt.upsi.edu.my; 7 Department of Electrical, Electronic & Systems Engineering, Faculty of Engineering & Built Environment, The National University of Malaysia, Bangi 43600, Selangor, Malaysia; huda.abdullah@ukm.edu.my; 8 School of Civil Engineering, Faculty of Engineering, Universiti Teknologi Malaysia, Skudai 81310, Johor, Malaysia; mhafizputeh@utm.my; 9 Faculty of Environmental Science, National University of Laos, Dongdok Campus, Vientiane P.O. Box 7322, Laos; oulavanhnoi@gmail.com

**Keywords:** PVDF hollow fiber membrane, triethyl phosphate, less toxic solvent, polyethylene glycol, air gap, contact angle, membrane technology

## Abstract

Existing toxic solvents in the manufacturing of polymeric membranes have been raising concerns due to the risks of exposure to health and the environment. Furthermore, the lower tensile strength of the membrane renders these membranes unable to endure greater pressure during water treatment. To sustain a healthier ecosystem, fabrication of polyvinylidene fluoride (PVDF) hollow fiber membrane using a less toxic solvent, triethyl phosphate (TEP), with a lower molecular weight polyethylene glycol (PEG 400) (0–3 wt.%) additive were experimentally demonstrated via a phase inversion-based spinning technique at various air gap (10, 20 and 30 cm). Membrane with 2 wt.% of PEG 400 exhibited the desired ultrafiltration asymmetric morphology, while 3 wt.% PEG 400 resulting microfiltration. The surface roughness, porosity, and water flux performance increased as the loading of PEG 400 increased. The mechanical properties and contact angle of the fabricated membrane were influenced by the air gap where 20 cm indicate 2.91 MPa and 84.72°, respectively, leading to a stronger tensile and hydrophilicity surface. Lower toxicity TEP as a solvent helped in increasing the tensile properties of the membrane as well as producing an eco-friendly membrane towards creating a sustainable environment. The comprehensive investigation in this study may present a novel composition for the robust structure of polymeric hollow fiber membrane that is suitable in membrane technology.

## 1. Introduction

Nowadays, membrane technologies are known as a leading method for addressing a separation process in separating liquid or gas [1]. As a result of the excellent features of membrane technology, namely a great separation system, saving energy operation [2], as well as the alternative for conventional methods, it serves as a demand clean technology for water treatment. Basically, membrane technology performance is correlated with several known parameters including membrane material, pore size, and type of effluent to be treated. Membranes can be classified into four different pore sizes which are microfiltration (MF), ultrafiltration (UF), nanofiltration (NF), and reverse osmosis (RO). The pore size of MF was stated between 0.1 to 10 µm and can be applied to surface waters and groundwater. MF also can be operated at 0.1–3 bar pressure. UF possessed a pore size ranging from 0.01 to 0.1 µm and can be operated at pressure 0.5–10 bar. In addition, UF has the capabilities to retain larger organic macromolecules. Besides, the pore size of NF typically varies from 0.0002–0.002 µm that falling between reverse osmosis membrane and UF membrane as well as have the ability in the removal of microorganisms, turbidity, and hardness of the water. While RO has a pore size between 0.0001–0.001 µm which is capable of the removal of total dissolved solids (TDS) [3].

Polymer materials such as polyvinylidene fluoride (PVDF), polyethersulfone (PES), polysulfone (PSF), polypropylene (PP) and polyethylene (PE) have been widely implemented so far in fabrication of membranes for water treatment. The polymeric membrane can bring privileges such as better flexibility, selectivity, low operation cost, and easy preparation [4,5]. Among these polymeric materials, PVDF is favored over other polymer materials for its outstanding capability to meet the vast demand on account of its good chemical resistance, stronger mechanical strength [6], and superior solubility in a variety of organic solvents.

Organic solvents namely N,N-dimethylacetamide (DMAc), N,N-dimethylformamide (DMF) and N-methyl pyrrolidone (NMP) were widely utilized to dissolve the PVDF in membrane fabrication. Unfortunately, these solvents exhibit toxicity that may cause serious health and environmental impacts. As can be seen through Material Safety Data Sheet (MSDS) by Sigma Aldrich chemical company, the Regulation (EC) No 1272/2008 labelled these compounds as tremendously toxic and harmful. Table S1 reported that DMAc, DMF and NMP may damage the unborn children and devastate the fertility [7,8]. Replacement or substitution towards other less or non-toxic solvents is quite challenging for human protection and environment sustainability [9].

Triethyl phosphate (TEP) is known as an eco-friendly solvent due to its reduced toxicity content, it is also not a carcinogenic, teratogenic, or mutagenic as reported in MSDS [8] in Table S1. Moreover, TEP offers a suitable replacement for other toxic solvents in polymeric membranes’ fabrication which can prevent the workers from serious health risks [10]. In addition, the efficiently replacement of hazardous solvents with TEP for fabrication of polymeric membrane also been confirmed by the blooming number of scientific papers [7,8,10,11,12,13,14,15]. Abed et al. [11] explained that the use of TEP helps in increasing the tensile strength of polymeric membrane and thus, it can withstand a strong pressure and resistance. Furthermore, interconnected pores in a membrane can also be developed by utilization of TEP [11]. As mentioned by Chang et al. [7], TEP has been used as a solvent for PVDF hollow fiber membrane’s fabrication via phase inversion. The literature stated that the PVDF/TEP system requires less non-solvent to induce phase inversion due to the weaker TEP solvent. Although TEP is a preferred low toxicity solvent, however it encounters a weaker solvent ability to dissolve the polymer as compared with the hazardous one as well as a denser membrane without formation of finger-like structure. In addition, PVDF exhibits a hydrophobic nature which may encounter a fouling issue during a water treatment.

To minimize the denser membrane problem that can lower the water flux performance as well as fouling issue, surface modification is needed to obtain a hydrophilic PVDF membrane. The added value of additives in polymeric membrane fabrication may lead to the better properties of membrane such as more porous structure, increase in finger-like length, larger pore size and enhance the membrane’s performance [16]. Among a variety of additives that been used in polymeric membrane’s fabrication, namely polyethylene glycol (PEG), polyvinylpyrrolidone (PVP), and lithium chloride (LiCl) [17], PEG is favored where it can give the optimum performance based on flux permeation stated by Aminudin et al. [16]. PEG is a low-cost additive and found to be easily dissolves in water and organic solvents as well as enhancing the membrane permeability [18]. Besides, PEG has a capability to produce a finger-like structure of membrane by reducing the thermodynamic stability in polymer dope solution [19] as well producing hydrophilic nature of polymeric membrane. PEG came from lower (<1000) to higher (>1000) molecular weight. According to Singh et al. [20] high molecular weight of PEG tends to produce a hydrophobic nature of membrane as it is considered as a thickening agent. While low molecular weight of PEG known as hygroscopic where it tends to absorb the moisture and mobile which help in penetrating deeply for formation of porous media [20]. Varying the air gap during the fabrication process also act as a modification technique that affect the properties of produced membrane.

In this study, TEP and DMAc were mixed to avoid the solubility issue of PVDF as TEP is a weak solvent. TEP was still used in higher weight percentage than DMAc to minimize the toxicity and health risk problem raised by DMAc. Additionally, several loading of lower molecular weight of PEG 400 acted as additive in polymer dope solution as a pore former and enhance the hydrophilicity of polymeric membrane. Hollow fiber membrane configuration was chosen due to the higher surface area exhibited compared to flat sheet configuration. Spinning air gaps at 10, 20 and 30 cm were chosen to investigate the changes properties of membrane. UF membrane was produced based on widely used for macromolecules separation from aqueous solution and this type of membrane reveals an important application which can provide high retentions of proteins especially for separation of biological solution. Hence, bovine serum albumin (BSA) was chosen as a solute to investigate the membrane separation efficiency. Therefore, a novel modified composition of 50% TEP with different composition of DMAc and PEG 400 with different air gap for PVDF hollow fiber membrane fabrication using dry-wet spinning technique in this study produce a robust characteristic of polymeric membrane with stronger tensile strength as well an excellent performance for water flux and BSA rejection.

## 2. Materials and Methods

### 2.1. Materials

Polyvinylidene fluoride (PVDF, Kynar 760 pellets) was provided by Solvay Specialty Polymers (Solvay, Brussels, Belgium) that act as polymer material. Solvents namely N,N-dimethylacetamide (DMAc) and triethyl phosphate (TEP) were purchased from Sigma Aldrich (St. Louis, MO, USA) and Merck (Kenilworth, NJ, USA), respectively. It is a colorless liquid with lower molecular weight polyethylene glycol (PEG 400) that acts as additive was provided by Evergreen Engineering & Resources (Selangor, Malaysia). Bovine serum albumin (BSA, MW: 67 kDa, Sigma Aldrich, St. Louis, MO, USA) was used for membrane’s rejection study.

### 2.2. Fabrication of Single Layer PVDF Hollow Fiber Membrane

#### 2.2.1. Preparation of Polymer Dope Solution

Moisture of PVDF pellets was removed by drying the PVDF pellets inside the vacuum oven at 60 °C for 24 h. Certain composition of TEP and DMAc was mixed and stirred at 240 rpm inside a Schott bottle (Schott, Mainz, Germany) with a temperature of 80 °C until homogenous. PVDF pellets with composition of 15 wt.% [21] was added into the solution and stirred under same condition until completely dissolved. Different loadings of PEG 400 were added into the mixture solution and stirred for another 24 h until homogenous. The compositions of TEP/DMAc/PEG400 for single layer PVDF hollow fiber membrane fabrication was revealed in Table 1.

#### 2.2.2. Dry-Wet Spinning Technique

**Figure 1:**
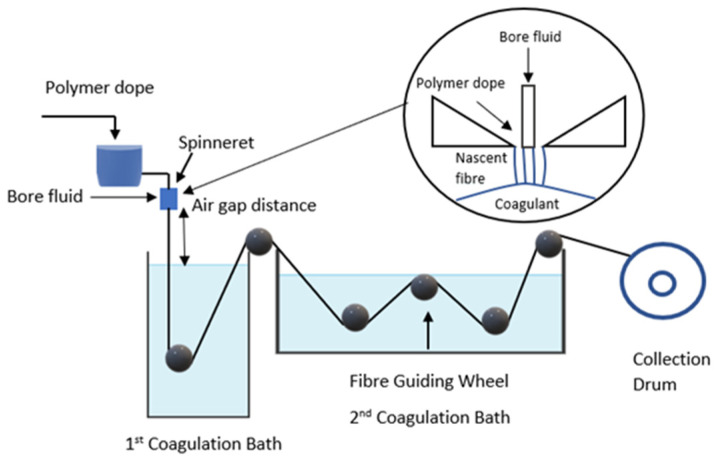
Polymeric hollow fiber membrane preparation by dry-wet spinning system.

Normal tube and double orifice spinneret were used in dry-wet spinning technique for fabrication of polymeric hollow fiber membrane configuration. Dope solution was degassed inside the sonicator bath (Ultrasonic cleaner, DC-150H, Delta Ultrasonic, Taipei, Taiwan) for 30 min to remove bubbles. Then, the dope solution was poured into the dope reservoir and spinning procedure was started according to the conditions shown in Table 2. The dry-wet spinning system is shown in Figure 1. Phase inversion process take placed when dope solution reaching into coagulation bath containing non-solvent for solidification. After that, as-spun hollow fiber was collected from the collector and immersed into a deionized water tank for 24 h to extract the residual diluent. Afterwards, the as-spun hollow fiber membrane was immersed into 50% of ethyl alcohol for 1 h for post treatment. The as-spun hollow fiber was soaked into 100% ethyl alcohol for another 1 h to prevent shrinkage effect of membrane. Finally, the single layer PVDF hollow fiber membrane was dried at room temperature for 1 day.

### 2.3. Physical-Chemical Characterization

The viscosity of different dope solutions containing different additive loading was investigated using a viscometer (Model: BROOK FIELD, Middleboro, MA, USA) at 20 rpm speed of spindle rotation with torque value of 50% or shear rate of 34 s^−1^. The morphology of the cross section of PVDF hollow fiber membrane (HFM) was determined through the use of a scanning electron Microscopy (SEM) (Model: TM3000, Hitachi). PVDF HFM was coated with platinum under vacuum to prevent from charging effect and ensure the micrograph is clear. The magnification used was 60×, 600× and 5K×. Meanwhile, the porosity and pore size distribution of PVDF HFM was analyzed using mercury intrusion porosimeter (MIP) (Model: AutoPore IV Series, Micromeritics, Norcross, GA, USA). The optimum pressure was applied to measure the pore size distribution of PVDF HFM. The roughness surface of PVDF HFM was investigated by analyzing it using atomic force microscopy (AFM) (Model: SE-100 Park System, Suwon, Korea). The surface of PVDF HFM was located in horizontal plane on the object stage and the tested area was scanned. The mechanical behavior of the fiber membrane was determined via a tensile test. Tensile test of PVDF HFM was done by loading a 50 mm sample at 500 N load cell (Model: Zwick/Roell, Ulm, Germany) with loading rate at 10 mm/min. Both end of PVDF HFM was gripped and pulled for elongation test. Tensile strength was calculated using Equation (1) [21]:

(1) $$\mathrm{Tensile}\;\mathrm{strength}\;\left(\mathrm{Pa}\right)=\frac{\mathrm{Load}\;\mathrm{at}\;\mathrm{break}\;\left(N\right)}{\mathrm{Cross}\;\mathrm{sectional}\;\mathrm{area}\;\left(m^{2}\right)}\;$$

The hydrophilic or hydrophobic nature of PVDF HFM was tested by dropping 2 µL of deionized water as a contact liquid on the membrane’s surface and measured by contact angle goniometer (Model: OCA15EC, Dataphysics, Filderstadt, Germany). Results were obtained by taking at least eight measurement points of one sample.

### 2.4. Pure Water Flux and BSA Rejection Performance

Water permeation analysis was conducted using crossflow permeation cell by pumping deionized water as feed across the membrane while allowing the permeate to flow out through lumen side of PVDF HFM. Three sample membranes were put together inside an adapter by gluing only one end of membrane. The performance analysis was carried out by 8 cm length of PVDF HFM with 1 bar pressure at 25 °C. The water flux was calculated using Equation (2) as follows:

(2) $$F=\frac{V}{A\;\times \;t}$$

where V is a permeate volume (L) collected. A is an exposed membrane filtration area (m^2^) while t (h) is time.

The performance of membrane for BSA filtration was determined by using 500 ppm BSA as a feed. BSA of 500 ppm was prepared by transferring 0.5g of BSA powder (67 kDa) into 1000 mL of volumetric flask and deionized water was added to the mark. Then, the flask was inverted to dissolve the BSA powder to obtain the homogenous solution. The BSA rejection of membrane was determined using Equation (3) below:

(3) $$R\;=\left(\frac{C_{0}-C_{1}}{C_{0}}\right)-1\;$$

where, C_0_ is the initial absorbance value of feed solution while C_1_ is the absorbance value of permeate. BSA content was conducted using a UV-Vis spectrophotometer (DR5000, HACH, Loveland, CO, USA) at wavelength of 282 nm by performing at 1 bar pressure at room temperature [22].

## 3. Results and Discussion

### 3.1. Viscosity of Dope Solution

**Figure 2:**
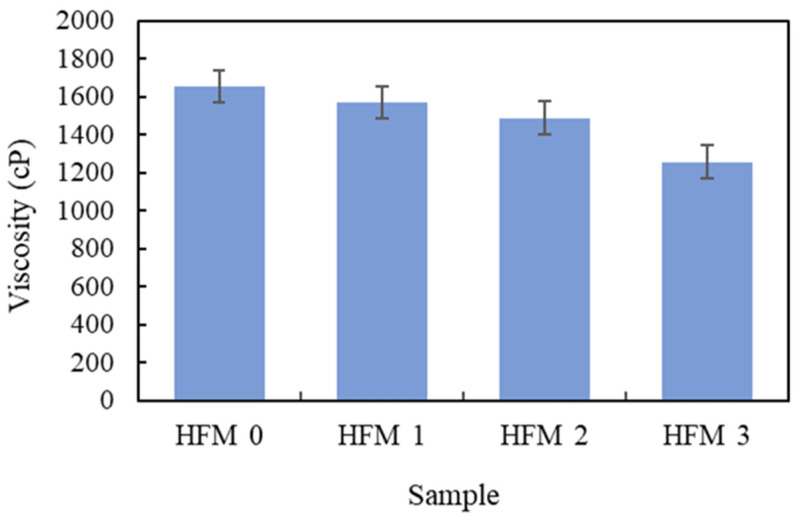
Viscosity of PVDF dope solution with 0, 1, 2 and 3 wt.% of PEG 400 at 20 rpm with torque value of 50% or shear rate of 34 s^−1^ (3 readings for each dope solution).

The maximum viscosity was measured at a chosen spindle at a speed range of 1–100 rpm. Every speed gave a different torque value. The viscosity value of the PVDF suspension was determined at different PEG 400 loadings at a speed range of 1100 rpm. To obtain a valid viscosity measurement, the torque value must be between 10% to 100% [23]. The critical viscosity values of PVDF suspension with 0 wt.%, 1 wt.%, 2 wt.% and 3 wt.% of PEG 400 were 1655 cP, 1572 cP, 1490 cP and 1258 cP, respectively, at 20 rpm with a torque value of 50% or shear rate of 34 s^−1^. As the speed is increased further, the viscosity value was unreadable which may be due to the reason that speed does not fit to the viscosity of the sample (23). The higher the torque value (>10%), the better is the accuracy of viscosity value on full scale range (FSR) (23). The viscosity is decreased by increasing the PEG 400 loading as shown in Figure 2. This could be due to the structure of lower molecular weight of PEG 400 in liquid form where it tends to reduce the viscosity when increasing the loading. According to Li et al. [24], flat PVDF membranes successfully prepared with composition of 15 wt.% PVDF, 60 wt.% TEP, 40 wt.% DMAc and 5 wt.% PEG 200. The viscosity of the prepared membrane at a shear rate of 10 s^−1^ is 7310 cP. The higher viscosity shown could be due to the stronger solvent power for PVDF on account of lower solubility parameter difference.

A similar trend was reported by Adam et al. [25], that the critical viscosity value for fabricated HFM was detected at 30 s^−1^. The viscosity determined at this shear rate was considered a threshold value for the formation of a finger-like structure in membrane morphology. Furthermore, the molecular weight of PEG 400 also influences the viscosity of the dope suspension. Since PEG 400 indicates the lower molecular weight of the pore former, it tends to reduce the viscosity of the dope suspension as well as produce a homogenous solution. Compared with the literature studied by Plisko et al. [26], the use of high molecular weight of PEG from 6000 g/mol and onwards, the formation of a homogenous solution is possible only at a certain range of temperature. Furthermore, the viscosity of dope solution was also increased by increasing the molecular weight of PEG, thus, increasing the turbidity of solution. Dzinun et al. [27] and Kamaludin et al. [21] utilized high molecular weight of additive namely PEG 6000 as the additive in PVDF dope solution, however, it exhibits a typical high viscosity of dope solution and the formation of a spongy membrane.

### 3.2. Morphology of PVDF HFM

**Figure 3:**
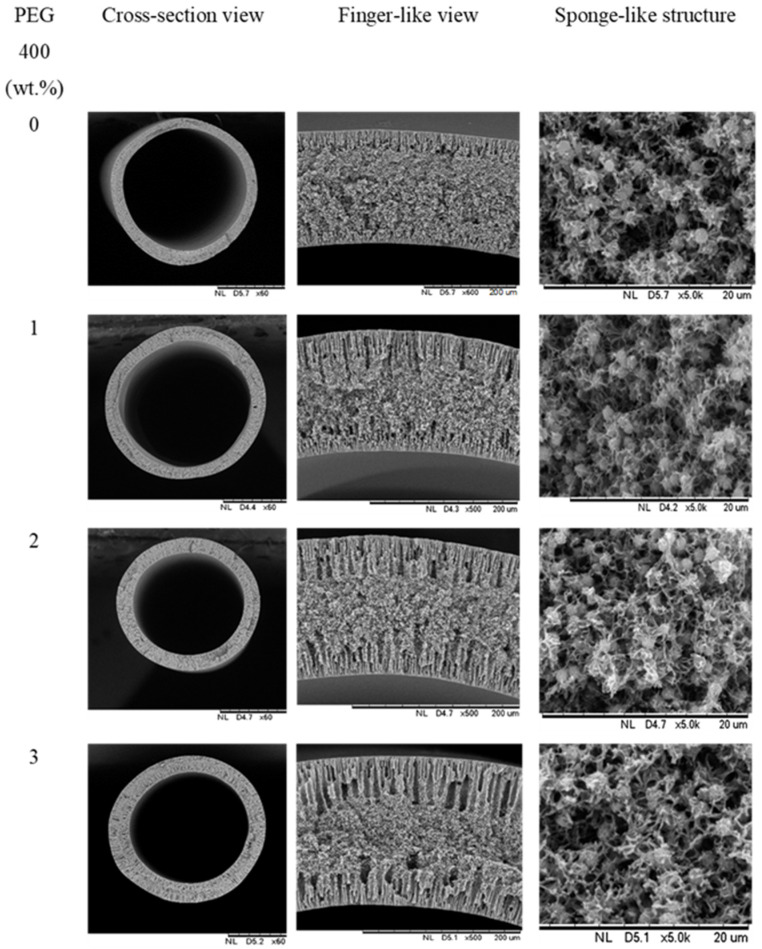
Morphology of PVDF HFM at 0, 1, 2 and 3 wt.% PEG 400 loading with 10 cm air gap.

The morphology of the cross section of PVDF HFM can be seen from SEM images shown in Figure 3. As shown in Figure 3, the finger-like structure was developed at the inner and outer layer of all samples, while the sponge-like structure developed at the intermediate layer. Additionally, a spongy structure at the intermediate layer can be known as a sandwich-like structure that is experienced during the phase inversion process. That structure was developed due to the suspension-coagulant interface instability [21]. The spongy structure may result from a slow precipitation rate whereas the creation of finger-like or porous structure was the result of the high precipitation rate [28] from the intrusion of the bore fluid and coagulation bath in the inner and outer surface, respectively. Different loadings of additive used in membrane manufacturing could result in the formation of different structures of produced hollow fiber membranes [15]. Moreover, the interaction of the solvents and non-solvent during membrane solidification impacts the developed morphology, whereas, the affinity of solvents affects the exchange rate of the solvent in the coagulation bath [29]. In most cases, instantaneous demixing results in the formation of a porous structure while formation of lower porous structure is formed by slow demixing [30]. Basically, low miscibility between polymer and non-solvent results in the repulsion of polymer chains at diffusion points of non-solvent molecules when the dope solution is immersed into the coagulation bath [31]. It results in the formation of a nuclei of polymer-poor phase in water molecules diffusion direction.

Moreover, the formation of interconnected pores was observed in all membrane’s samples due to the presence of TEP as a solvent. Abed et al. [11] also reported that the use of TEP as solvent helps in producing interconnected pores for better water flux performance. Previous literature has also reported that the interconnected pores of flat sheet membrane was successfully produced by using TEP as solvent in PVDF casting. TEP is considered a weaker solvent as it exhibits a weaker mutual affinity with non-solvent compared with DMAc [10]. As can be seen in micrograph, the higher the PEG 400 loading (0–3 wt.%), the longer the finger-like structure developed. By maintaining the spinning air gap at 10 cm, the average of finger-like structure developed by HFM 0, HFM 1, HFM 2 and HFM 3 was 23.2 µm, 27.3 µm, 33.5 µm and 69.73 µm, respectively. This was on account of the role of higher PEG 400 loading that present higher amount of OH group. Hydroxyl group will attract more water during fabrication process and hence, creating more pores as well as developing finger-like structures [32]. Furthermore, as the viscosity decreased when increasing the PEG loading, the TEP/DMAc-water exchange tended to be delayed, thus enhancing the formation of porous membrane [8]. Longer finger-like structures were crucial in determining the higher permeability which is vital for water treatment process [25].

**Figure 4:**
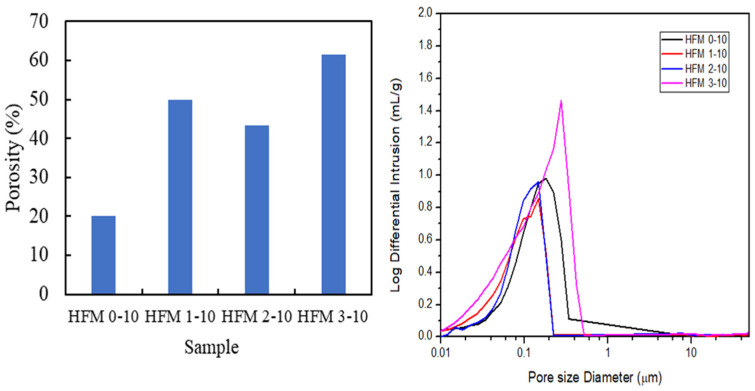
Porosity and pore size distribution of PVDF HFM at 0, 1, 2 and 3 wt.% loading of PEG 400.

Porosity and pore size distribution of PVDF HFM at different loading of PEG 400 was revealed in Figure 4. Mercury intrusion porosimetry analysis is capable of determining the porosity degree of the produced membrane. As PEG 400 loading increased from 0 wt.% to 3 wt.%, the porosity also increased from 20.17% to 61.61%. However, the porosity of PVDF HFM at 2 wt.% PEG 400 slightly reduced and this slight shift could be due to strong interactions between PEG and PVDF via hydrogen bonding [33]. The excessive amount of PEG 400 at 3 wt.% could act as pore-forming agent resulting in larger pore size. The increment of PEG 400 loading increased the porosity of the membranes produced with the porosity of 61.61% when 3 wt.% PEG 400 was used, which indicates that the produced HFM was composed of large pores that are sufficiently porous for a polymeric membrane. Hence, the porous membrane will be beneficial for possessing higher water permeability [34].

Similar findings were reported in literature by Wang et al. [35]: that the mean pore size of flat poly(vinylidene fluoride-hexafluoropropylene) (PVDF-HFP) porous membranes were measured by using the mercury intrusion porosimetry (MIP) method. According to Milescu et al. [36], this method explains the phenomenon of “nonwetting” liquids in capillary not being able to be absorbed by the pores of a solid, and requiring external pressure. The volume of pores in different sizes can be obtained by intruding the mercury into the sample material with each pressure change. In addition, this method only shows the accessible interconnected pores while the closed pores are incompressible. The pressure of applied mercury is inversely proportional to the size of pores, where large pores need to be penetrated using lower pressure while small pores need to be penetrated using greater pressure. Pore size can be determine precisely using this method since the volume of mercury can be determined accurately [37].

MIP is useful in determining the pore size distribution of sponge-like structures and finger-like structures. However, the pore size distribution from finger-like structures do not influence the pore size distribution of the peak as shown by the graph in Figure 4. The single broad peak revealed by each sample membrane indicates that the membranes were composed of symmetrical sponge-like structures that are dominant, and represented the uniform pore formation throughout the HFM in this study [34]. From the graph, HFM 3-10 exhibits intense pore size distribution at a separative layer of membrane from the average sponge-like structure formed between 0.1 to 0.5 µm, referring to the microfiltration membrane. While pore size distribution of HFM 2-10, HFM 1-10 and HFM 0-10 is reduced at 0.1 µm which were identified as an ultrafiltration membrane. In addition, on increasing the PEG 400 loading, the mercury intrusion intensity also increased. This indicates that the pore size of PVDF HFM became larger when high loading of PEG 400 is added. This phenomenon occurred due to the presence of larger OH groups inside PEG 400 which attracts water (bore fluid) during the fabrication process, producing larger pores. Since HFM 2 is found to be the best composition as an ultrafiltration membrane and exhibits a longer finger-like structure compared to HFM 0, HFM 1 and HFM 3, dope solution with 2 wt.% PEG loading was used in the fabrication of membrane at different spinning air gap.

**Figure 5:**
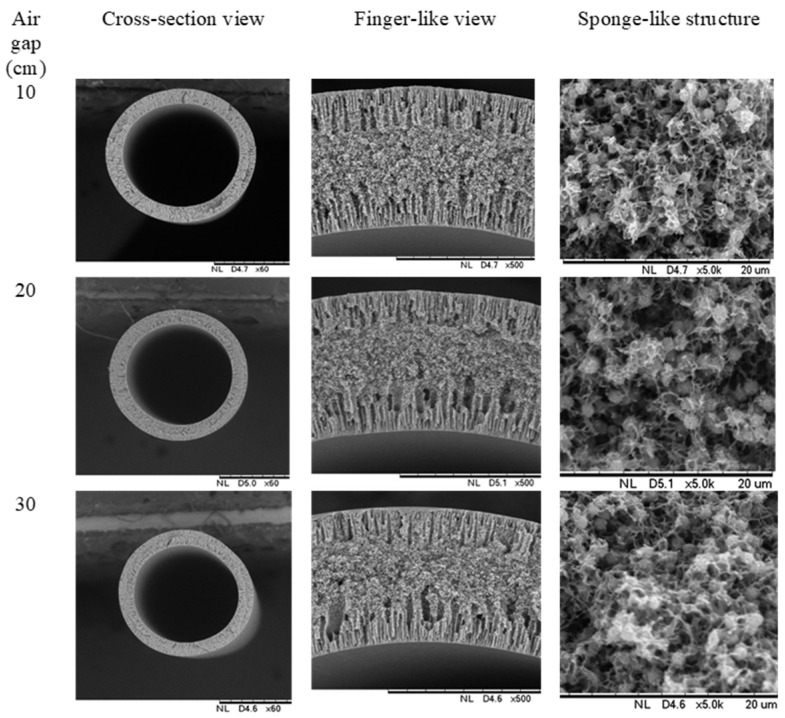
Morphology of PVDF HFM at 2 wt.% PEG 400 loading with different air gap.

Spinning parameters, namely the air gap, was also revealed to have a significant effect on overall morphology of the produced fiber membrane. As can be seen in Figure 5, the finger-like structure was increased when a higher air gap was applied during the spinning procedure. This could be due to the increasing air gap that will influence the phase inversion process. A higher air gap will provide more time for bore fluid to intrude from inside, before the membrane can solidify once it enters the coagulation bath. Hence, creating longer finger-like structures at the inner side of the membrane. However, as the air gap increases to 30 cm, the sponge structure becomes looser and finger-like voids appear beneath the fiber outer layer, hence reducing the finger-like length. This occurrence was on account of the phase separation of dope in drying process. As the dope solution passes through higher air gap, it comes to longer contact with air and faces weak phase inversion. Thus, more water vapor in air permeates into dope solution and functioning as non-solvent additives as well the stretch stress on the membrane under the gravity helps in producing larger pore size [38]. Besides, the outer diameter of PVDF HFM was decreased with increase in air gap. Outer diameter of HFM 2-10, HFM 2-20 and HFM 2-30 exhibit 1510 µm, 1300 µm and 1230 µm as the air gap increased from 10 cm to 30 cm, respectively. Furthermore, wall thickness of membrane also decreased when increasing the air gap to 30 cm. The wall thickness decreased from 360 µm to 280 µm for HFM 2-10 – HFM 2-30 as the same can be observed in Table 3. The phenomenon of decreasing diameter and thickness of hollow fiber membrane with increasing air gap could be based on spin line stresses experienced by nascent fiber before it solidifies completely in water. A further stretching of fiber resulted from higher elongational stress at higher air gap during phase separation before dope solution reaches non-solvent coagulation bath [11]. As a result, the higher the air gap, the higher the stretching of fiber membrane is experienced thus producing thinner outer diameter of membrane. This result was also supported with the polyether sulfone/polyvinyl alcohol hollow fiber membrane produced by Ahmad et al. [39] that produced smaller diameter hollow fibers on increasing air gap.

**Figure 6:**
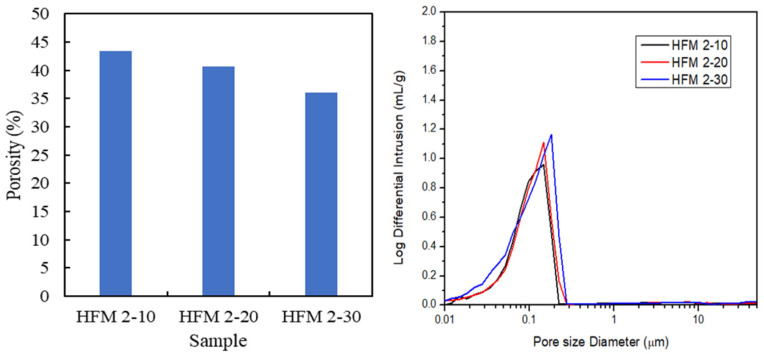
Porosity and pore size distribution of PVDF HFM 2 at different air gap.

Figure 6 depicts the porosity and pore size distribution of 2 wt.% PEG 400 PVDF HFM at different air gap. On increasing the air gap from 10 cm to 30 cm, porosity decreased from 43.39% to 40.67% while pore size at separative layer of PVDF HFM from average sponge-like structure tends to increase, larger than 0.1 µm which referred to microfiltration membrane. This phenomenon could be due to the stretching of nascent fiber during fabrication process at higher spinning air gap where pore might be elongated, hence, increasing the pore size.

### 3.3. Surface Roughness of PVDF HFM

**Figure 7:**
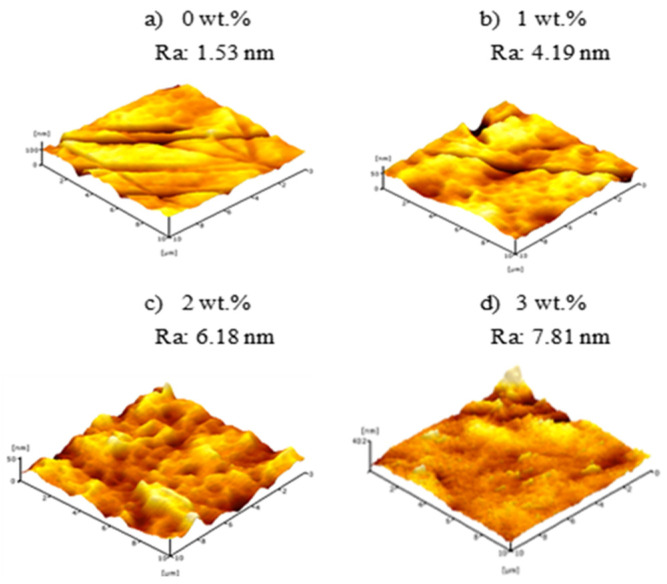
Surface roughness of PVDF HFM at different loading of PEG 400.

**Figure 8:**
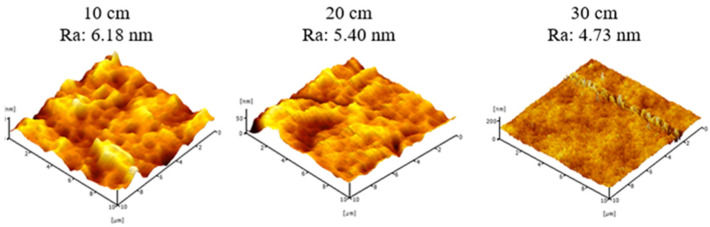
Surface roughness of PVDF HFM 2 at different air gap.

Figure 7 presents 3D micrograph of surface roughness (Ra) of PVDF HFM prepared at different additive loadings. The AFM images can be clearly observed that the surface roughness of membrane has no significant difference and show slightly increase on increasing the loading of PEG 400. The Ra value of HFM 0-10, HFM 1-10, HFM 2-10 and HFM 3-10 were 1.53 nm, 4.19 nm, 6.18 nm and 7.81 nm, respectively. This could be due to the development of pores as the loading of PEG 400 is increased which also affects the surface roughness of membrane. Despite this, surface roughness of PVDF HFM 2 tends to decrease from 6.18 nm to 4.73 nm when air gap increased from 10 cm to 30 cm as can be seen in Figure 8. This might be on account of longer contact time of dope suspension exposed to air at higher air gap before it completely solidifies in coagulation bath. Thus, the stretching phenomenon can cause the nascent fiber possessed a smooth surface. Surface roughness of PVDF HFM was crucial in determining suitable support for the deposition procedure. For the water treatment process, higher surface roughness of the membrane will have high selectivity as support to be coated with material that can provide higher available surface area and expected to minimize the leaching issue [40]. Kuvarega et. al. [41] explained the risk of nanoparticle leaching from a membrane substrate during high pressure application or continuous operation. Srinivasan et al. [42] supported the stronger attachment of nanoparticle on the support was needed to ensure the reusability of the material for water treatment.

### 3.4. Mechanical Strength and Wettability of PVDF HFM

**Figure 9:**
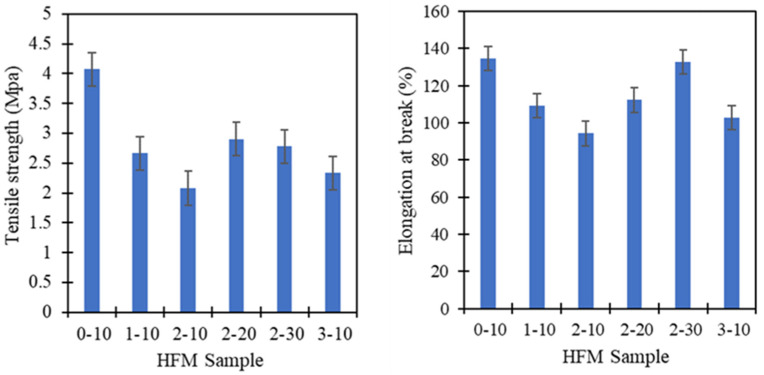
Tensile strength and elongation at break of PVDF HFM at different PEG 400 loading and air gap for 5 samples each membrane.

**Figure 10:**
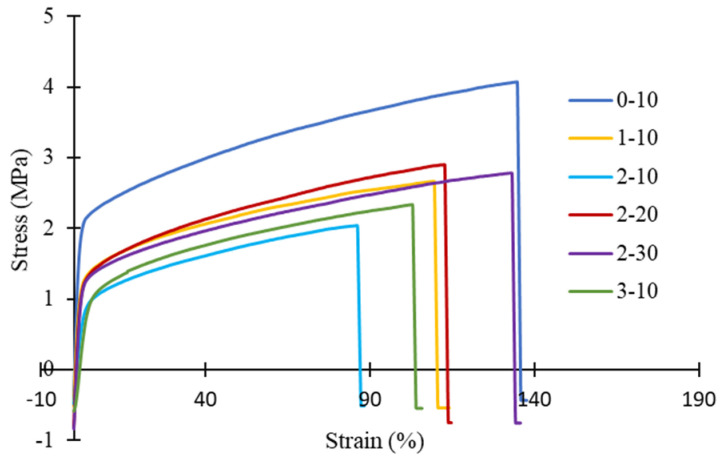
The stress-strain curve of PVDF HFM at different PEG 400 loading and air gap.

Figure 9 summarizes the results of tensile strength and elongation at break for PVDF HFM at various PEG 400 loading and air gap. While, Table 4 details the tensile properties of all the PVDF samples. Tensile strength of PVDF HFM deteriorated due to increasing of PEG 400 loading as additive. This condition could be due to the larger pores created at high loading of additive which makes it easy to stretch and break. However, tensile strength and elongation at break increased dramatically as air gap increased. It could be due to the stretching of nascent fiber that forces the fiber to be elongated during fabrication procedure, hence increasing the elongation at break of PVDF HFM 2 at higher air gap. Tensile strength of HFM 0-10 was the strongest as compared with other samples due to the presence of smaller pore size. Moreover, PVDF HFM produced without addition of PEG 400 exhibits stronger tensile strength due to the shorter finger-like length produced with spongy structure at intermediate space of membrane. Stunningly, all samples exhibit stronger tensile strength which is higher than 2 MPa as compared to previous literature written by Dzinun et al. [27] that only exhibit 1.82 MPa for single layer of PVDF hollow fiber membrane fabricated by using DMAc only. This could be due to the effect of TEP help in producing interconnected pores inside the membrane [11], thus, producing a stronger membrane. It is expected that it can withstand high pressure during treatment. The elongation at break of HFM 0-10 also higher at 134.56% which the membrane can stretched longer before it breaks. Figure 10 gives a stress strain curve for all PVDF samples, demonstrating the higher percentage elongation at break shows by HFM 0-10.

**Figure 11:**
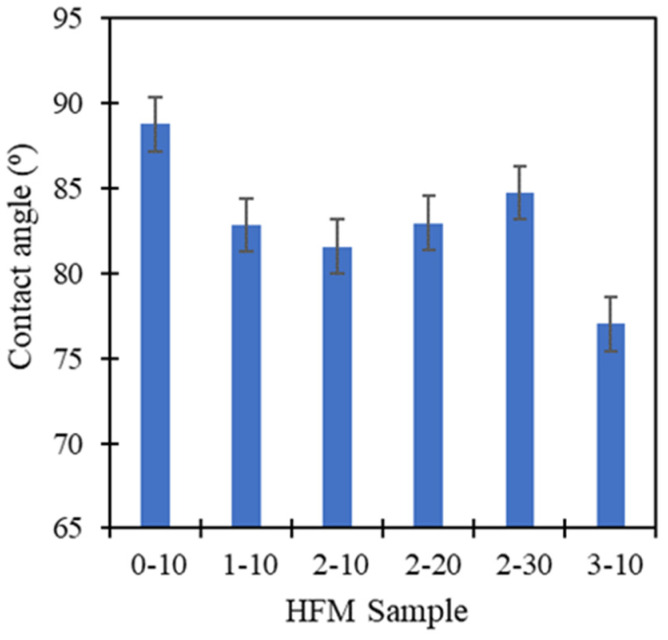
Average contact angle value of PVDF HFM at different PEG 400 loading and air gap at 8 points of membrane.

Figure 11 depicts the contact angle value of PVDF HFM decreased from 88.74° to 77.01° as loading of PEG 400 increased from 0 to 3 wt.%. Water contact angle depends on the effect of chemical structure (polar or non-polar) and surface roughness of polymer surface [43]. As PEG 400 is hydrophilic polymer, polar groups easily interact with water molecules, thus, influence the water contact angle. Moreover, lower molecular weight of PEG 400 tends to have more polar (-OH) groups resulting lower total surface energy. As a result, lower water contact angle observed can be confirmed and the hydrophilicity nature was achieved with increasing the loading of PEG 400 [43]. This could be due to the entrapment of PEG 400 in the membrane as well as increasing the surface roughness that increases the surface area [40] causing the membrane to exhibit hydrophilic characteristics. The wetting improvement could be explained by an effective PEG 400 used as additive in producing hydrophilic membrane. Basically, hydrophilicity shows the interaction between membrane and foulants such as hydrogen bonding, dipole interaction, Van Der Waals interaction and electrostatic effect. HFM 3-10 exhibited high surface tension and possessed an ability to form hydrogen bonds with water due to the higher OH groups in PEG 400, thus, develops a water layer between membrane and solution [27]. As can be seen through Figure 11, the contact angle value of PVDF HFM 2 increased from 81.56° to 84.72° as the air gap increased from 10 cm to 30 cm. This result could be due to the reducing of the surface roughness of the membrane’s surface so that it reduced the surface area of sample. Thus, a smooth surface of the fiber membrane was produced, lending itself to a hydrophobic nature. Additionally, the wetting nature of the membrane possessed significant parameters for preventing a fouling problem. The fouling issue has been widely discussed in water treatment as it play as important role and can decrease the water productivity [44]. The higher contact angle corresponded to the hydrophobicity of membrane which the particles may clog inside the pores. Thus, reducing the water permeability.

### 3.5. Water Flux and BSA Performance

**Figure 12:**
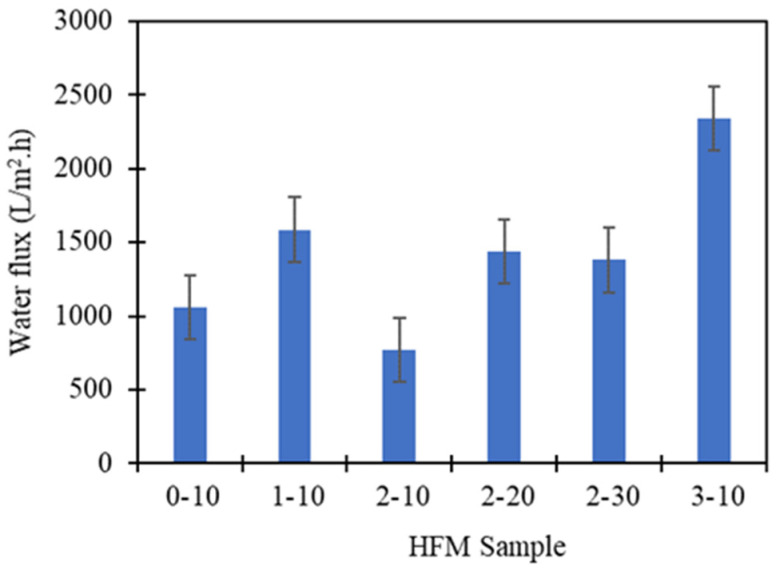
Water flux performance of PVDF HFM at different PEG 400 loading and air gap for 3 samples each membrane.

All the hollow fiber membranes were prepared with different loadings of PEG 400 additive and different spinning air gaps were analysed for the pure water flux analysis according to equation [45], as shown in Figure 12. When the higher loading of additive was added, the PVDF HFM showed a significant improvement in the performance of water flux. HFM 0-10 without PEG 400 shows low water flux at 1060.54 L/m^2^ h due to the shorter finger-like formation as well as smaller pores exhibited. When PEG 400 loading increased to 1 wt.%, the flux improved to 1585.85 L/m^2^ h. However, it declined when 2 wt.% of additives were added. It can be supported with the lower porosity exhibited which is in the agreement with the pure water flux that could be due to the non-homogenous dope solution. Furthermore, the entanglement between PEG 400 molecules and polymer chains was enhanced which leads to a relatively denser outer skin, hence the water flow channel inside the fiber membrane became smaller. This statement can be supported by Feng et al. [33] who found that pore size distribution and pure water flux shows a similar result with this study from the effect of PEG 400 loading. Lower loading of PEG 400 causes strong hydrogen bonding and enhanced entanglement between PEG 400 and polymer while higher loading of PEG 400 act as pore former in producing larger pore size in produced membrane. Despite this, when the additive increased to 3 wt.%, the water flux dramatically increased at 2338.88 L/m^2^ h. This could be explained by the longer finger-like structure and development of larger pores which act as microfiltration membrane due to effect of PEG 400 as a pore former, as mentioned in Section 3.2. When the finger-like structure is longer and the pores produced are larger, the water easily flows into the membrane’s lumen, which improves the water flux performance. These results agree with the previous paper reported by Plisko et al. [26] which explained the membrane obtained using lower molecular weight of additive (PEG 400) which exhibited highest water flux value. Furthermore, Wu et al. [45] reported that the highest water flux produced at 47.4 L/m^2^ h when PEG 400 used in fabrication of polyamide-polysulfone composite forward osmosis membrane. However, the water flux result reported by Wu et al. [46] was still lower compared to this study which could be due to the mixed NMP/DMF solvents used in membrane fabrication. The addition of PEG 400 causes the casting solutions thermodynamically less stable due to weak non-solvent PEG-400 in NMP/DMF system. Previous literature explained by Nawi et al. [47], when 3 wt.% of PEG with higher molecular weight of 20 kDa was used in PVDF membrane fabrication, the clean water permeability achieve only 750 L/m^2^ h bar which the performance is poor compared to this study that used lower molecular weight of PEG 400.

The same improvement also can be seen by varying the air gap from 10 cm to 30 cm. HFM 2-10 exhibit 768.12 L/m^2^ h water flux at 10 cm air gap which is lower than the 20 cm and 30 cm air gap that exhibits 1436.31 L/m^2^ h and 1379.83 L/m^2^ h, respectively. This can be on account of the longer finger-like structure developed at a higher air gap. The higher the air gap, the longer the time taken for bore fluid to intrude inside the fiber membrane, performing longer finger-like structures before solidifying inside the coagulation bath, thus, enhancing the water flux performance. However, the pure water flux of PVDF HFM fabricated at 30 cm air gap is slightly reduced due to the shorter finger-like length produced at higher air gap because of the phase separation of dope in the drying process [38] that has been explained in Section 3.2, therefore, 20 cm air gap shows optimum condition for outstanding pure water flux performance. Previous literature reported that the water flux performance of PVDF flat sheet membrane achieved is 24 ± 1.3 L/m^2^. h when 2 wt.% of PEG 400 was added [31], which is lower than this study. The comparison can be seen through the differences in the solvents used. In previous literature DMAc was used as solvent [31] as compared to this study where mixed DMAc/TEP solvents in membrane fabrication were used. The addition of TEP as solvent in membrane fabrication helps to produce bicontinuous morphology with interconnected porous membrane structure which has been explained in Section 3.2 that support the enhanced water flux produced [11]. The wall thickness of membrane also played a significant role in determining the flux performance. As the air gap increased, wall thickness was decreased as already described in Section 3.2. The reducing wall thickness provides lower resistance for water to flow thus resulting higher flux.

**Figure 13:**
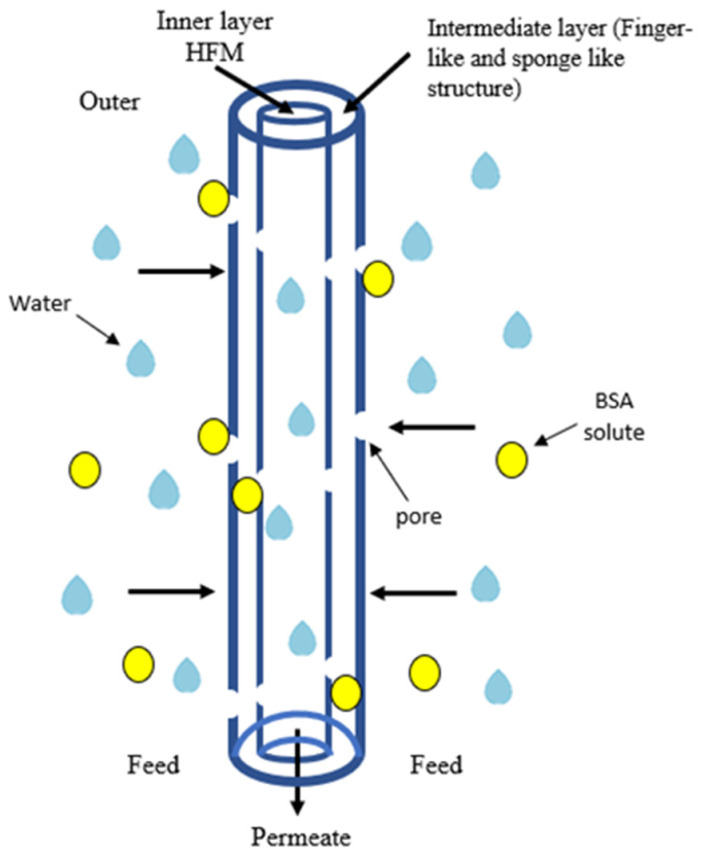
Mechanism of BSA rejection using PVDF HFM in cross flow permeation cell.

**Figure 14:**
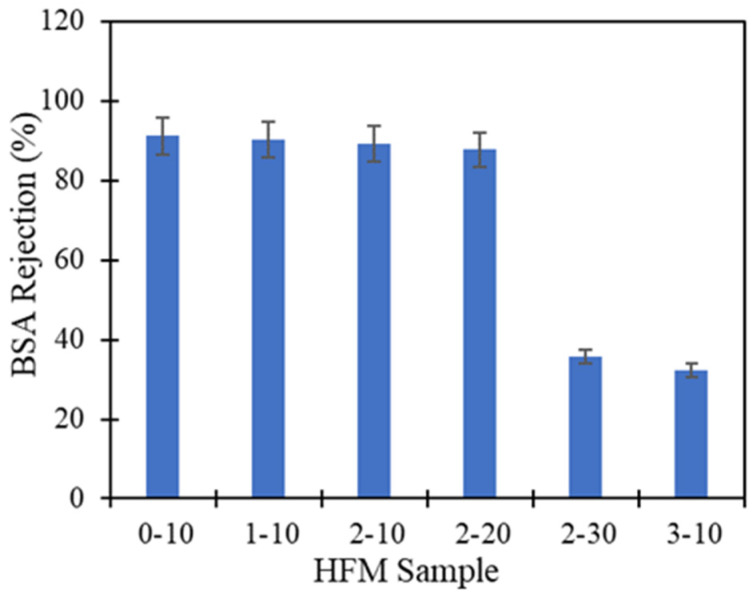
BSA Rejection performance of PVDF HFM at different PEG 400 loading and air gap for 3 samples each membrane.

Figure 13 indicated the mechanism of BSA rejection using PVDF HFM in cross flow permeation cell. BSA solution was pumped as feed across the membrane while allowing the permeate to flow out through lumen side of PVDF HFM. The larger size of BSA solute with size of 0.0101 µm at pH range 7–4.7 [48] was retained at membrane pore and inner finger-like while allowing the clean water pass through it. The rejection capability of BSA solution of all the PVDF HFM samples were done and shown in Figure 14. As can be seen from the figure, HFM 0-10 achieved highest rejection capability at 92.1% while HFM 3-10 exhibit lowest BSA rejection at 32.32%. HFM 3-10 behaves as microfiltration membrane because the pore size was bigger (0.1 µm), which causes smaller 0.01 µm size of albumin particles slipping through the pores into the permeate section. Thus, lowering the rejection capability. HFM 0-10 act as ultrafiltration membrane with smaller pore size ranging between 0.01–0.1 µm and consequently it is difficult for BSA to penetrate through them. This can be proved when HFM 0-10 exhibit excellent rejection capability > 90%. Previous literature reported the PVDF/Fe^3+^/Cu^2+^ hollow fiber membranes exhibit 91.6% BSA rejection by adding 1.5 wt% PEG 400 as pore former [49]. Moreover, it is stated by Ma et al. [50], that flat sheet PVDF membrane fabricated by using 17 wt% PVDF, DMAc and 5 wt% of PEG 400 exhibit only 80% BSA rejection performance which is lower than this study. As higher loading of PEG 400 was used, the larger the pore sizes of membrane are achieved, thus, lowering the rejection capability. Besides, when higher air gap was applied during spinning procedure, the BSA rejection capability is decreased as the pore size of membrane increased, as can be seen only 35.75% BSA been rejected by HFM 2-30 whereas the small albumin particles can easily slip through the larger pores. In addition, contact angle value also influences the rejection capability. As the air gap increased, the water contact angle also increased thus leading to hydrophobicity. Hydrophobicity can cause the fouling issue along with reduced rejection capability. The comparison of water flux performance and BSA rejection obtained in this study with literature was revealed in Table 5.

## 4. Conclusions

PVDF hollow fiber membrane fabricated at different loadings of additive and different spinning air gaps have been successfully prepared via a dry wet spinning technique. From the overall analysis, it can be concluded that, the higher the PEG 400 loading, the better the morphology produced for outstanding water flux performance. PVDF HFM fabricated at 3 wt.% loading of PEG 400 demonstrated as microfiltration membrane with higher porosity, larger pores, and longer finger-like structure as well as showing an excellent water flux performance at 2338.88 L/m^2^ h. However, tensile strength and elongation at break of PVDF HFM declined as higher PEG 400 loading was added which indicated that the sample cannot support higher load when being stretched. HFM 2 can be indicated as an optimum membrane that possessed ultrafiltration membrane which is suitable for its use in separation of smaller particles and the produced fiber membrane exhibits well developed morphology. By varying the air gap to 20 cm as a suitable air gap results in a high porosity membrane, stronger tensile strength, higher surface roughness as well as an excellent water flux and BSA rejection performance. Higher surface roughness of HFM 2-20 at 5.40 nm was qualified to be used as a good support membrane for coating, which is expected to minimize the leaching issue during water treatment. Furthermore, lower toxicity of TEP can be a problem solver in minimizing the existing toxic solvents and also helps in producing outstanding mechanical strength of fabricated membrane. Stronger tensile of HFM 2-20 at 2.91 MPa also provides an advantage if used in high pressure conditions where it might withstand greater resistance. Overall, the ultrafiltration membrane of HFM 2-20 is expected to be a selective membrane for future water treatment with various applications by maintaining the sustainability of nature.

## Figures and Tables

**Table 1:** Composition of TEP/DMAc/PEG 400 for fabrication of PVDF hollow fiber membrane.

Sample	PVDF (wt.%)	TEP (wt.%)	DMAc (wt.%)	PEG 400 (wt.%)
HFM 0	15	50	35	0
HFM 1	15	50	34	1
HFM 2	15	50	33	2
HFM 3	15	50	32	3

**Table 2:** Parameter for fabrication of PVDF hollow fiber membrane using dry-wet spinning technique.

Sample Name	Dope Flow Rate (rpm)	Bore Fluid Type	Bore Fluid Flow Rate (mL/min)	Coagulation Bath	Spinneret Dimension (mm)	Air Gap (cm)
HFM 0-10	26	Water	8	water	0.8/1.2	10
HFM 1-10						10
HFM 2-10						10
HFM 2-20						20
HFM 2-30						30
HFM 3-10						10

**Table 3:** Outer diameter and thickness of PVDF HFM at different air gap.

Sample	Air-Gap (cm)	Outer Diameter (µm)	Thickness (µm)
HFM 2-10	10	1510	360
HFM 2-20	20	1300	305
HFM 2-30	30	1230	280

**Table 4:** Average tensile test results, giving tensile strength (σ_max_) and elongation at break (ε_b_).

PVDF Sample	σ_max_ Tensile Strength (MPa)	ε_b_ Elongation at Break (%)
0-10	4.08 ± 0.1	134.65 ± 0.8
1-10	2.66 ± 0.8	109.18 ± 1.0
2-10	2.08 ± 0.1	94.3 ± 4.3
2-20	2.91 ± 0.3	112.28 ± 2.8
2-30	2.78 ± 0.1	132.74 ± 1.0
3-10	2.34 ± 0.3	102.74 ± 1.1

**Table 5:** Comparison of water flux performance and BSA rejection obtained in this study with literature.

Sample	Type of Solvent	PEG 400 Loading (wt.%)	Air Gap (cm)	Water Flux (L/m^2^ h)	BSA Rejection (%)	Reference
HFM 0-10	TEP/DMAc	0	10	1060.54	91.2	This study
HFM 1-10	TEP/DMAc	1	10	1585.85	90.3	This study
Flat sheet PA/PSF	NMP/DMF	0	-	28.6	-	[45]
Flat sheet PA/PSF	NMP/DMF	3	-	36.5	-	[45]
Flat sheet PA/PSF	NMP/DMF	6	-	47.4	-	[45]
Flat sheet PA/PSF	NMP/DMF	9	-	39.9	-	[45]
Flat sheet PVDF	DMAc	2	-	24 ± 1.3	-	[31]
Flat sheet PVDF	DMAc	4	-	30 ± 2.5	-	[31]
Hollow PVDF/Fe^3+^/Cu^2+^	DMAc	1.5	12	35	91.6	[47]
Flat sheet PVDF	DMAc	5	-	-	80	[48]

## Data Availability

Not applicable.

## Supplementary Materials

The following are available online at https://www.mdpi.com/article/10.3390/membranes11110843/s1, Table S1: Hazards statements of DMAc, DMF, NMP and TEP according to Regulation (EC) No 1272/2008.
