# Supplementary material for: Fabrication of High Performance PVDF Hollow Fiber Membrane Using Less Toxic Solvent at Different Additive Loading and Air Gap
Source: Membranes (Basel). 2021 Oct 29;11(11):843. doi: 10.3390/membranes11110843 (PMC8622764; doi:10.3390/membranes11110843)
Supplement: Supplementary file 1 [file membranes-11-00843-s001.zip › membranes-1418775-supplementary.pdf]

membranes

MDPI

Supplementary Material

Fabrication of High Performance PVDF Hollow Fiber Membrane using Less Toxic Solvent at Different Additive Loading and Air Gap

Hazirah Syahirah Zakria[1,2], Mohd Hafiz Dzarfan Othman[1,2,*], Siti Hamimah Sheikh Abdul Kadir[3,], Roziana Kamaludin[1,2], Asim Jilani[4], Muhammad Firdaus Omar[5], Suriani Abu Bakar[6], Juhana Jaafar[1,2], Mukhlis A. Rahman[1,2], Huda Abdullah[7], Mohd Hafiz Puteh[8], Oulavanh Sinsamphanh[9], Muhammad Ayub[1,2],**

1 Advanced Membrane Technology Research Centre (AMTEC), Universiti Teknologi Malaysia, 81310 Skudai, Johor, Malaysia; hazirahzakria@gmail.com (H.S.Z.); roziana.kamaludin7@gmail.com (R.K.); juhana@petroleum.utm.my (J.J.); mukhlis@petroleum.utm.my (M.A.R.); ayub1977@graduate.utm.my (M.A.)
2 School of Chemical and Energy Engineering, Faculty of Engineering, Universiti Teknologi Malaysia (UTM), 81310 Skudai, Johor, Malaysia;
3 Institute of Pathology, Laboratory and Forensics (I-PPerForM), Faculty of Medicine, Universiti Teknologi MARA (UiTM), Cawangan Selangor, 47000 Sungai Buloh, Selangor, Malaysia
4 Center of Nanotechnology, King Abdul-Aziz University, 21589 Jeddah, Saudi Arabia; asim.jilane@gmail.com
5 Scientific Computing and Instrumentation (SCnI) Research Group, Physics Department, Faculty of Science, Universiti Teknologi Malaysia, 81310 Skudai, Johor, Malaysia; firdausomar@utm.my
6 Nanotechnology Research Centre,Faculty of Science and Mathematics, Universiti Pendidikan Sultan Idris, 35900 Tanjung Malim, Perak, Malaysia; suriani@fsmt.upsi.edu.my
7 Department of Electrical, Electronic & Systems Engineering, Faculty of Engineering & Built Environment, The National University of Malaysia 43600 Bangi, Selangor, Malaysia; huda.abdullah@ukm.edu.my
8 School of Civil Engineering, Faculty of Engineering, Universiti Teknologi Malaysia, 81310 Skudai, Johor, Malaysia; mhafizputeh@utm.my
9 Faculty of Environmental Science, National University of Laos, Dongdok, Campus, P.O.Box: 7322, Vientiane, LAO PDR; oulavanhnoi@gmail.com.
* Correspondence: hafiz@petroleum.utm.my
** Co-correspondence: sitih587@gmail.com

Table S1. Hazards statements of DMAc, DMF, NMP and TEP according to Regulation (EC) No 1272/2008 [7,8].

Solvent	Regulation (EC) No 1272/2008		Diseases
	**Hazard codes**	**Hazard statements**	
DMAc	H312 + H332	Harmful when skin contacted or inhaled	Pancreatis
	H319	Causes serious eye irritation	
	H360D	May damage the unborn child	
DMF	H226	Flammable liquid and vapour	Hepatitis, Testicular cancer
	H312 + H332	Harmful when skin contacted or inhaled	
	H319	Causes serious eye irritation	
	H360D	May damage the unborn child	

NMP	H315 + H319	Causes skin and serious eye irritation	Miscarriage, Light birth weight
	H335	May cause respiratory irritation	
	H360D	May damage the unborn child	
TEP	H302	Harmful if swallowed	-
	H319	Causes serious eye irritation	
